# O.4.5-11 The association between health literacy and objectively measured physical activity in Norwegian young adult students

**DOI:** 10.1093/eurpub/ckad133.219

**Published:** 2023-09-11

**Authors:** Tania Milena Salazar, Ann-Katrin Grotle

**Affiliations:** Western Norway University of Applied Sciences, Norway; salazar_tania@hotmail.com; Western Norway University of Applied Sciences, Norway; salazar_tania@hotmail.com

## Abstract

**Background:**

Insufficient physical activity (PA) is a significant public health challenge, with a drastic decline in activity levels observed in young adulthood likely contributing to increases in cardiometabolic risk factors with age. Recent research suggests that health literacy (HL) may be an important modifiable factor associated with reduced physical activity levels and poor health outcomes in elderly and disease-prone populations. However, limited research has focused on young adulthood when the drastic decline in PA is observed. Moreover, most previous studies have only assessed self-reported PA, which has limited accuracy. Therefore, the purpose of this study was to assess the relationship between HL and objectively measured PA levels in healthy young adults.

**Method:**

28 young adult (18-25 years) college students participated in the study (men: n = 7, women: n = 21). PA was measured objectively and subjectively using an accelerometer (Actigraph GT3X+) and the IPAQ questionnaire, respectively. The HLS19 instrument was used to assess HL, which was developed within “HLS19 – the International Health Literacy Population Survey 2019-2021” of M-POHL. Participants were categorized as having low or high health literacy based on the classification defined in the Norwegian HLS-19 report (2021). Additionally, each response was assigned a value (1-4) to calculate a summative health literacy point score, with a higher summative value indicating greater health literacy.

**Results:**

In our cohort, 60% (n = 17) of the participants were categorized as having low and 39% (n = 11) as having high health literacy. There was no group difference in objective and subjective PA levels between those with low and high HL (all comparisons: p > 0.05). However, we observed a significant positive correlation between health literacy (point score) and objectively measured moderate to high-intensity PA (r = 0.52, p = 0.002). Likewise, there was a significant correlation between health literacy (point score) and self-reported total PA (r = 0.34, p = 0.03).

**Conclusion:**

This is the first study to show a positive relationship between HL and PA in healthy young adult students, highlighting the potential role of HL in modulating PA in this population. Thus, further research is needed to determine whether targeting HL could effectively increase PA levels in young adults.

